# Implementation of best-evidence rehabilitation for hip, knee and hand osteoarthritis in Africa: A cause for concern

**DOI:** 10.1016/j.ocarto.2025.100606

**Published:** 2025-03-29

**Authors:** Monique M. Keller, Beatrice Sankah

**Affiliations:** a Department of Physiotherapy, School of Therapeutic Sciences, Faculty of Health Sciences, University of the Witwatersrand, 7 York Street, Parktown, 2193, Gauteng, South Africa; b Department of Sports and Exercise Science, School of Allied Health Sciences, University of Cape Coast, Ghana

**Keywords:** Osteoarthritis, Physical activity, Physiotherapists, Rehabilitation, Evidence-based practice, Africa

## Abstract

**Importance:**

Evidence-based practice recommendations and guidelines highlight the benefits of exercise, physical activity, weight management, and education for self-management, which are effective in the management of osteoarthritis (OA). However, the uptake among African health professionals remain poor. The objective of this commentary is to highlight the disparity between current best-evidence rehabilitation practices and African health professionals’ practices amidst the high prevalence of OA.

**Observations:**

The World Health Organization emphasizes the importance of physical activity in light of the rising OA prevalence in the ageing population, with the burden of comorbid disease negatively impacting the ageing population's quality of life. The concern is that even though the physical activity directive from the World Health Organisation clearly outlines the physical activity targets and best-evidence recommendations and guidelines for managing patients with OA, African health professionals' uptake remain low, potentially to the disadvantage of patients.

**Conclusions and relevance:**

Increasing OA prevalence with subpar rehabilitation may result in lower quality of life and function for individuals diagnosed with hip, knee, hand and other joint OA, leading to a substantial financial burden on the individual, family, community, stakeholders and governmental levels. Development of African context-specific evidence-based rehabilitation guidelines and improved training on current guidelines by universities and professional societies are needed. Policy makers prioritizing OA as a pertinent burden of disease is lacking in Africa. A multidisciplinary team approach is needed where early referral to physiotherapists is emphasized.

## 1 Introduction

The World Health Organization (WHO) designated 2021–2030 as the decade of healthy ageing due to the urgent need to address prevalent musculoskeletal conditions, including osteoarthritis (OA), which is one of the leading causes of disability and pain in Africa [1,3]. Osteoarthritis impacts independence and quality of life [2] as well as human functioning [3]. The pain, stiffness, decreased functioning and quality of life resulting from OA impact the above 60-year adult population and can start younger than 50 years [4]. In 2020, knee OA was the most prevalent age-standardised area of OA globally, followed by hand OA [3].

In Africa, it is reported that individuals diagnosed with OA often have multiple comorbidities posing as risk factors [1]; hence, the life years lived with disability, taking into account a global aged standardised knee OA prevalence of 3.8 ​%, is a staggering 17.1 million years. Whittaker et al. [5] stated that individuals with OA have an increased risk of comorbidities, with approximately 59–87 ​% having at least one chronic condition, with 31 ​% of individuals living with OA having five or more chronic conditions [6]. The problem is that Africa significantly contributes to the global OA prevalence, leaving individuals who not only are diagnosed with OA but frequently have other chronic conditions with unmet needs and treatment burden [7].

This commentary aims to raise awareness about the prevalence of OA in Africa, sharing the best-evidence hand, knee and hip rehabilitation OA management research, highlighting African research that shows decreased uptake of best-evidence OA rehabilitation implementation among physiotherapists and other health professionals. Three critical management determinants of OA are emphasized. An urgent call to action for policymakers, governments, professional societies, and universities in Africa is made to eliminate the barriers to implementation to improve the quality of life for individuals diagnosed with OA.

## 2 Prevalence of osteoarthritis in Africa

Steinmetz et al. [3] conducted a “Global Burden of Disease 2021″ study to project the national, regional and global burden of disease projections to provide evidence towards the WHO directive to improve healthy ageing. In 2020, the prevalence of OA globally with age-standardised prevalence for females was 8058.9 per 100 ​000 (UI 7251.9–8867.9) at 95 ​% and for males 5780.1 per 100 ​000 (5217.8–6341.2). With increased age, the total prevalence of OA increases. The 70 years and above group 38418.9 per 100 ​000 (34471.4–42302.7) compared to 23237.2 per 100 ​000 (20390.8–26108.6) among 50 to 69 ages [3].

When considering the prevalence of knee, hand, hip and other joint OA in Sub-Saharan Africa, it is noteworthy that Sub-Saharan Africa comprises sub-regions and countries per region, as presented in Table 1 below. Added to Table 1 are the prevalence statistics for knee, hand, hip, and other joint OA in Sub-Saharan Africa and sub-regions.

**Table 1:** Sub-Saharan Africa sub-regions knee, hand, hip and other joint osteoarthritis prevalence per 100,000 [3].

Sub-Saharan Africa sub-regions and countries	Knee	Hand	Hip	Other joints
**Sub-Saharan Africa (combined)**	3654·2 (3237·5–4155·8)	1913·2 (1475·0–2418·1)	373·8 (286·1–473·4)	696·5 (557·8–909·6)
**Central sub-Saharan Africa** (Angola, Central African Republic, Congo (Brazzaville), Democratic Republic of Congo, Equatorial Guinea, and Gabon)	3435·1 (3038·9–3883·4)	2009·4 (1552·8–2570·1)	346·4 (263·6–444·1)	660·8 (527·0–869·7)
**Eastern sub-Saharan Africa** (Burundi, Comoros, Djibouti, Eritrea, Ethiopia, Kenya, Madagascar, Malawi, Mozambique, Rwanda, Somalia, South Sudan, Uganda, Tanzania, and Zambia)	3453·8 (3058·5–3915·6)	1807·5 (1393·1–2286·5)	367·4 (282·0–465·9)	676·4 (542·8–887·2)
**Southern sub-Saharan Africa** (Botswana, Eswatini, Lesotho, Namibia, Zimbabwe, and South Africa)	3924·9 (3496·6–4486·1)	2746·8 (2134·5–3441·4)	494·8 (376·1–633·6)	777·5 (618·1–1018·8)
**Western sub-Saharan Africa** (Benin, Burkina Faso, Cabo Verde, Cameroon, Chad, Côte d'Ivoire, The Gambia, Ghana, Guinea, Guinea-Bissau, Liberia, Mali, Mauritania, Niger, Nigeria, São Tomé and Príncipe, Senegal, Sierra Leone and Togo)	3804·8 (3366·8–4358·5)	1719·2 (1320·6–2173·5)	350·6 (268·7–441·4)	699·5 (560·1–911·8)

Southern sub-Saharan Africa had the lowest OA prevalence globally, 2419.7 per 100 ​000 (2184.7–2674.4). However, compared to the other Southern sub-Saharan African countries, South Africa, has the highest prevalence of knee, hand, hip and other joints [3].

What is even more concerning is that compared to the global prevalence of hand OA, 2226.1 per 100 ​000 (1719.7–2802.8), including previously mentioned Southern sub-Saharan African countries, South Africa has an alarming hand OA prevalence 2999.6 per 100 ​000 (2332.9–3757.3). The same counts for hip OA prevalence in South Africa being 517.7 per 100 ​000 (392.4–663.8) compared to the global prevalence 417.7 (314.7–532.7) [3]. In Western sub-Saharan Africa, Nigeria has higher prevalence rates for knee: 3807.3 (3368.5–4347.9), hand: 1934.4 (1485.6–2441.8), hip: 349.6 (267.5–438.5), other: 709.9 (565.1–926.9). Hence, it is important to deliver best-evidence OA rehabilitation towards prevention and disease progression globally but more urgently in Africa, especially countries like South Africa and Nigeria, to allow individuals affected by OA to have a better quality of life and healthy aging in future decades [3].

## 3 Evidence-based management practices for hip, knee and hand osteoarthritis in Africa and internationally

When considering the management of individuals presenting with hip, knee and OA or a combination of joints affected by OA, multiple studies highlight similarities across best-evidence or evidence-based clinical practice guidelines. Examples of some recommended management interventions used internationally are exercise, weight management, education for self-management and physical activity to name a few [6,[8], [9], [10], [11], [12], [13]].

The updated European League Against Rheumatism (EULAR) recommendations for managing hand OA [12] recommends that target management focus on reducing pain and stiffness, optimising hand function, and enhancing quality of life, activity levels, and participation. All individuals diagnosed with hand OA must receive comprehensive information about the nature of the condition, its progression, strategies for self-management, and available treatment options. Similar to knee OA, hand OA management should be tailored to the individual, including a multidisciplinary approach where appropriate within the context of the management. Additionally, hand OA recommendations also emphasise education and training on activity pacing, use of assistive devices, and the use of ergonomic principles. Prescribed exercises should focus on improving muscle strength and hand function and reducing pain. Other best practice recommendations for hand OA management are strengthening, stretching and joint mobility exercises [14]. For individuals with thumb base OA, also known as carpometacarpal joint OA, orthoses are recommended to alleviate pain, with long-term use being encouraged [12].

Whittaker et al. [5], in a review of systematic reviews of rehabilitation treatments for OA, reported that the most effective rehabilitation treatments for OA include resistance training for all joints affected by OA, followed by strength training and aquatic exercises, with yoga being the most effective pain-reducing, non-surgical and non-pharmacological intervention.

Kolasinski et al. [11] developed a management guideline for hand, hip and knee OA in the “American College of Rheumatology/Arthritis Foundation Guideline” (ACR/AFG), and more recently, Moseng et al. [9] revised and provided an updated 2023 EULAR evidence-based recommendations for the non-pharmacological management of knee and hip OA. The above guidelines include recommendations for both multicomponent, multidisciplinary, and individualized management plans. Some management plans recommended for healthcare practitioners include education, information on the OA, self-management, and advice related to work. Other recommendations include exercise management with prescription, progression, dosages and mode of delivery adequately tailored to individual needs. Similarly, weight loss and maintaining a healthy weight are also advocated [11].

Bannuru et al. [6], in the OA Research Society International updated guidelines, recommended a land-based structured exercise programme with education about arthritis with or without weight management and dietary lifestyle considerations. Walking aids, assistive devices and footwear are inspected and correctly prescribed. Lifestyle adjustments through behaviour change techniques [15,16] are implemented to reach a healthy lifestyle [9]. The problem is that although best-evidence management practices and guidelines are available to effectively manage OA, current reported management approaches, especially in Africa, omit many of these recommendations [17].

After looking at international recommendations, African studies will now be presented. A South African primary care approach of land-based exercise programs, weight reduction strategies, use of appropriate assistive devices and a multidisciplinary approach that is individually tailored are recommended as non-pharmacological interventions for managing OA [18]. Education for self-management and physical activity are highlighted and shown to improve pain and outcomes experienced by individuals with OA. The recommended education is pain coping skills training [19]. Table 2 below shows the management practices, distinguishing between Africa and international recommended interventions.

**Table 2:** Recommended interventions for managing osteoarthritis in Africa and internationally.

Region	Interventions
African	Exercise, weight management, assistive devices, multidisciplinary approach [18]
African	Education for self-management, physical activity, multidisciplinary approach [19]
International	Aerobic exercise, resistance exercise, hydrotherapy, weight loss [11]
International	Multidisciplinary approach, patient-centered care, non-pharmacological strategies [9]
International	Structured exercise program, education about arthritis, weight management, lifestyle adjustments [6]

## 4 Uptake of best-evidence osteoarthritis rehabilitation management practices in Africa

Despite evidence-based treatment and OA management guidelines, the uptake of these is globally underutilised [20]. Africa has a low uptake of evidence-based management practices [20,21], consistent with the above statement, but with extensive barriers causing hindrances to community and primary health care settings [21].

In a cross-sectional survey completed by 104 Nigerian physiotherapists, only 16.30 ​% of participants had knowledge about clinical practice knee OA guidelines and 14.40 ​% adhered to guidelines in their treatments [21].

To improve the implementation of OA management programs, the Joint Effort Initiative international collaboration shared the top-ranked survey results topics that included behaviour change considerations as part of the training and education and future research to improve the uptake of physical activity, exercise and weight loss [22].

A qualitative study focused on exploring the rehabilitation needs and service delivery of patients with knee OA in rural Western Cape, South Africa [23]. Sixteen semi-structured interviews with 15 females and one male participant aged 67.5 (49–84) were conducted. Deductive analysis revealed three major themes identified, namely, “I would like to know more”, “There's not much support from the clinic” and “I don't feel myself anymore”. “I would like to know more” revealed a lack of condition and disease progression education led to perceptions that little can be done, whether daily activities affected their symptom progression and what treatment options are available. One participant stated “Okay, the doctor tells me they can't do anything about it. It is just a lifelong pain that you have to bear … I would like to know more. I've had it for so long now, more than 20 years … They told me the one was going to infect the other one later on. Is it true? (>20 years diagnosed)” [23].

“There's not much support from the clinic” revealed that treatments were predominantly pharmacological management with minimal mention of rehabilitation and pain management. A participant shared, “That's it. Only painkillers for my legs” (>20 years diagnosed) and “ … then they merely told me it's arthritis and prescribed painkillers. Then I didn't go back again (12 tears diagnosed)”. One participant received a comprehensive rehabilitation programme, and two received a dietician referral with an overall frustration with service delivery [23].

“I don't feel myself anymore” theme made it clear that participants experienced significant participation restrictions in their lives due to decreased mobility affecting their self-worth and mental well-being. A participant shared, “I have to sort of drag my legs when I have to do something. Like sweeping I still can't do … But because I'm alone, I have to do it … and it's difficult you know … you may be poor, my father used to say, but you don't need to be untidy. That's what I always remember. It's a really a struggle for me” (>20 years diagnosed).

The study highlights the barriers to socially inclusive OA management in a rural primary health care system, with inadequate human resources, continuity of care and referral systems [23] providing new insights on what is required to improve OA management in South Africa.

Eyles et al. [24], in their Joint Effort Initiative, aim to improve the implementation of best evidence OA programs globally that include low and middle-income countries. The panel discussion included South Africa, Nepal and Brazil and the main themes that emerged as barriers to best-evidence OA care were identified. Unaffordable, inequitable, unimportant, uncoordinated, and inexperienced. The results revealed that OA lacks recognition as a significant health issue, the fragmented healthcare systems, high costs of OA management, and the inexperience regarding knowledge and skill of health professionals [24]. Eyles et al. [24] provide value insights on improving OA management in low and middle-income countries such as South Africa.

In a Nigerian mixed method study including a cross-sectional survey and focus group, the physiotherapists working in the secondary and tertiary health facilities' knowledge, attitude and implementation of best-evidence therapeutic exercises for knee OA was investigated. Despite a fair understanding (81 ​%), knowledge (95.3 ​%) of evidence-based practices and its effectiveness, the physiotherapists had poor attitudes towards implementing the best-evidence practices [25].

## 5 Critical management determinants for osteoarthritis

### 5.1 Education and communication

Pain Neuroscience Education is a crucial component in the management of OA pain for individuals who live with pain due to OA. Pain often leads to the discontinuation of enjoyable hobbies and activities, as fear avoidance and altered gait patterns can diminish quality of life. However, a thorough understanding of OA flare-ups, pain physiology, and treatment options can be potentially transformative. Regarding pain, recent studies report symptom-structure discrepancies in the pain experience of people with hand OA [26]. For example, among the five hand OA phenotypes described according to radiographic hand OA burden, psychosocial burden and pain sensitisation, the phenotype with the least radiographic hand OA severity but more pain sensitisation reported more pain in hands than the phenotype with the most severe radiographic OA in the hands [26]. Hand OA pain experience is multifactorial because research shows that a higher burden of comorbidity, presence of back pain and depression are associated with greater pain severity and long-term pain [27]. Hence, it highlights the importance of considering comorbidities in managing people with hand OA. Therefore, for optimal clinical relevance, it is important to identify possible modifiable risk factors for pain so that management can be adjusted accordingly [26].

### 5.2 Behaviour change

Behaviour change is also highlighted as a critical determinant in OA management [9]. Willet et al. [15] evaluated the effectiveness of techniques of bahaviour change to increase physical activity adherence in physiotherapy interventions for individuals with lower limb OA. Willet et al. [15] identified the “behaviour change technique taxonomy (V1)" techniques as Michie et al. [16] developed in their consensus reporting study. Behavioural change techniques have high effectiveness in improving physical adherence in lower limb OA, and they include “patient-led goal setting”, “behavioural contract”, “social support”, “self-monitoring or behaviour”, “non-specific reward” [15]. Behaviour change strategies can improve the increased uptake of physical activity adherence [15], and physiotherapists are advised to use such behaviour change adherence tools.

### 5.3 Physical activity

World Health Organization [28] states that physical inactivity has been identified as the fourth leading risk factor for global mortality. The prevalence of physical inactivity is increasing in many countries, significantly impacting the incidence of non-communicable diseases and adversely affecting the overall health of populations worldwide. The WHO guideline, according the Bull [29], stipulates that for adults older than 18 years, the recommended physical activity is between 150 and 300 ​min per week of moderate intensity, or 75–150 ​min per week of vigorous-intensity physical activity, or a combination of moderate and vigorous intensity [29].

Individual assessments, personalised exercise prescriptions, and home exercise programmes can effectively be supplemented with follow-up group exercise classes in contexts with limited resources. These classes not only enhance the physical benefits of the exercise programme but also foster social interactions, motivation, and community bonds in solidarity in fighting the effects of OA.

## 6 Discussion

This commentary discussed the literature regarding the prevalence of OA in Africa and the best-evidence recommendation for hip, knee and hand OA management whilst highlighting the gaps in African research and the decreased uptake of best-evidence OA rehabilitation interventions among physiotherapists and other health professionals.

The commentary aimed to provide literature reports on the high prevalence of hand, hip and knee OA and the best-evidence rehabilitation for managing these conditions in Africa whilst also highlighting the gaps in research. For example, in a South African rural primary health care setting, qualitative findings reported the barriers and lack of socially inclusive OA management within the region [23]. A low-level evidence South African study recommended exercise, weight management, an individualized multidisciplinary approach and assistive device use [18], as seen in international studies [6,11,22]. A high level of evidence randomised controlled trial [19] concurred with international studies that physical activity [28,29] and education [6] are effective in managing OA [19]. The limited African evidence related to therapy interventions and guidelines for Africa further necessitates future research studies and the use of international best-evidence rehabilitation management.

Several studies are consistent with recommending as first line evidence exercise, education for self-management, physical activity and weight management as recommended guidelines [6,[8], [9], [10], [11], [12], [13]]. The benefit of exercises and structured, regular physical activity has been demonstrated for individuals who present with painful and symptomatic knee OA [30]. Fransen et al. [30] demonstrated in their systematic review that land-based exercises instantly improved physical function and reduced pain after the exercise programme. Twenty-two per cent of included studies showed a significant knee pain reduction for both the two- and six-month follow-up periods, and with 18.52 ​% of the study's physical function improvement over a more extended follow-up period [30].

Studies [9] also refer to individually tailored exercise programmes as essential for achieving positive health outcomes for individuals with OA, particularly in the context of additional chronic co-morbid conditions, and this may be challenging in the African context, with education, knowledge, and skill training necessary. Bannuru et al. [6] reported core elements in the updated OA Research Society International management guidelines for polyarticular, knee and hip OA with various comorbidities as land-based exercise programmes or load-bearing according to mode, dosage and progression [9] that should include components of aerobic, neuromuscular and/or balance, and strengthening exercises, and mind-body based exercises such as Yoga or Tai Chi for knee OA. Walking aids and hydrotherapy exercises were downgraded from core elements to treatments with positive effects due to financial restrictions in specific contexts. Not all [6] of the above are reported in other studies, perhaps due to a lack of evidence.

## 7 Recommendations

A plea for future research includes the development of evidence-based context-specific care in low-resource African settings [7]. An identified need includes contextually appropriate and accessible educational packages for patients to promote the uptake of evidence-based care [7].

Eyles et al. [24] highlighted the urgent need for South African policymakers to include OA in the burden of disease list and renewed discussions with the government and the South African Rheumatism and Arthritis Association to prioritise OA care. Universities and professional societies should include best-evidence OA management in the curricula and prioritise OA care. Recognition and inclusion of alternate, complementary medicine health practitioners and traditional healers in skills and OA education training [24].

African physiotherapists and other health professionals are encouraged to use best-evidence rehabilitation guidelines, including exercise, education, self-management, physical education, and weight loss in managing individuals diagnosed with OA.

The efficacy of exercise as a treatment modality depends on the appropriate type, prescription and progression of exercise regimes. As stated in the EULAR recommendations [9], exercise programmes should have clear prescriptions such as dosages and progression, and the individual affected by OAs needs and mode of exercise preferences should be considered and, hence, individually tailored.

## 8 Conclusion

African physiotherapists and other health professionals must use best-evidence rehabilitation management clinical practice recommendations and physical activity directives for patients diagnosed with any stage of OA, as prevention through behaviour change and exercise are imperative to enhancing patients' quality of life.

## Author contributions

The authors made substantial contributions to conception and design, manuscript draft writing and revisions as well as final approval of the manuscript.

## Declaration of Generative AI and AI-assisted technologies in the writing process

During the preparation of this work the author MK used Grammarly in order to improve the final language and readability. After using Grammarly the authors reviewed and edited the content as needed and takes full responsibility for the content of the publication.

## Role of the funding source

None funding source was involved in this manuscript.

## Declaration of competing interest

The authors declare no competing interests. The information presented is not from any institution or organisation but the authors' opinions.
